# Increasing incidence of malaria in children despite insecticide-treated bed nets and prompt anti-malarial therapy in Tororo, Uganda

**DOI:** 10.1186/1475-2875-11-435

**Published:** 2012-12-28

**Authors:** Prasanna Jagannathan, Mary K Muhindo, Abel Kakuru, Emmanuel Arinaitwe, Bryan Greenhouse, Jordan Tappero, Philip J Rosenthal, Frank Kaharuza, Moses R Kamya, Grant Dorsey

**Affiliations:** 1 Department of Medicine, San Francisco General Hospital, University of California, San Francisco, CA, USA; 2 Infectious Diseases Research Collaboration, Kampala, Uganda; 3 Global AIDS Program, Centers for Disease Control and Prevention, Atlanta, GA, USA; 4 Centers for Disease Control and Prevention, Kampala, Uganda; 5 Department of Medicine, Makerere University College of Health Sciences, Kampala, Uganda

**Keywords:** Malaria, *Plasmodium falciparum*, Immunity, Epidemiology

## Abstract

**Background:**

The burden of malaria has decreased in parts of Africa following the scaling up of control interventions. However, similar data are limited from high transmission settings.

**Methods:**

A cohort of 100 children, aged six weeks to 10 months of age, were enrolled in an area of high malaria transmission intensity and followed through 48 months of age. Children were given a long-lasting insecticide-treated bed net (LLIN) at enrolment and received all care, including monthly blood smears and treatment with artemisinin-based combination therapy (ACT) for uncomplicated malaria, at a dedicated clinic. The incidence of malaria was estimated by passive surveillance and associations between malaria incidence and age, calendar time and season were measured using generalized estimating equations.

**Results:**

Reported compliance with LLINs was 98% based on monthly routine evaluations. A total of 1,633 episodes of malaria were observed, with a median incidence of 5.3 per person-year (PPY). There were only six cases of complicated malaria, all single convulsions. Malaria incidence peaked at 6.5 PPY at 23 months of age before declining to 3.5 PPY at 48 months. After adjusting for age and season, the risk of malaria increased by 52% from 2008 to 2011 (RR 1.52, 95% CI 1.10-2.09). Asymptomatic parasitaemia was uncommon (monthly prevalence <10%) and rarely observed prior to 24 months of age.

**Conclusions:**

In Tororo, despite provision of LLINs and prompt treatment with ACT, the incidence of malaria is very high and appears to be rising. Additional malaria control interventions in high transmission settings are likely needed.

**Trial registration:**

Current Controlled Trials Identifier NCT00527800

## Background

Malaria causes more than 500 million clinical cases and is responsible for about one million deaths annually, mostly among African infants and young children [1]. Uganda bears a particularly large burden, having among the highest rates of transmission worldwide [2]. Within Uganda, there is significant heterogeneity of malaria transmission. In the most recent malaria indicator survey, from 2009, parasite prevalence among children under five years of age ranged from <5% in urban centres to >60% in rural settings [3].

As utilization of malaria control interventions, including long-lasting insecticide treated bed nets (LLINs), indoor residual spraying of insecticides, and prompt treatment with artemisinin-based combination therapy (ACT) has increased, many reports have shown a substantial drop in malaria transmission, malaria-associated hospitalizations, and malaria-associated deaths [1,4-8]. However, these findings have not been uniform across Africa [5,9,10]. One recent study showed a lack of decline in slide positivity rates and admissions for cerebral malaria from 2001–2010 in Malawi, and another showed an increase in malaria-related hospitalizations over a similar time period in Uganda [9,11]. These, and many other relevant recent studies, were retrospective in design, and so unable to provide reliable estimates of malaria incidence.

Considerable knowledge regarding the natural history of malaria, acquisition of anti-malarial immunity, and impact of interventions has been gained from longitudinal cohort studies. Beginning with Koch’s seminal studies in Papua New Guinea in the late 1800s, these studies have consistently revealed that, in endemic populations, the incidence and severity of malaria decreases considerably after the first years of life, with a corresponding rise in the prevalence of asymptomatic carriage of parasites [12-15]. Although widespread use of LLINs and ACT have been associated with declines in the incidence of malaria in many regions, there have been concerns over how decreasing exposure to malaria parasites may alter the natural history of disease, and possibly delay the acquisition of anti-malarial immunity [5,10,15]. To better understand the impact of LLINs and ACT on the natural history of malaria in a high endemicity setting, the incidence of malaria and prevalence of asymptomatic parasitaemia were evaluated in a cohort of children living in Tororo, Uganda.

## Methods

### Study site and participants

The Tororo Child Cohort (TCC) study was conducted in Tororo, a rural district in south-eastern Uganda with an entomological inoculation rate (EIR) estimated at 562 infective bites per person year (PPY) in 2002 [16]. Details of this study have been described elsewhere [17-20]. Briefly, convenience sampling was used to enroll children referred to a dedicated study clinic from an adjacent post-natal clinic at Tororo District Hospital. The sub-study described in this report included only children born to HIV-uninfected mothers who met the following enrolment criteria: 1) age six weeks to <10 months, 2) agreement to come to the study clinic for any febrile episode or other illness, 3) residence within a 30 km radius of the clinic, 4) absence of an active medical problem requiring in-patient evaluation at screening, and, 5) provision of informed consent. At enrolment, all study participants received an LLIN (Permanet).

### Follow-up of study participants

Subjects were followed for all medical problems at a dedicated study clinic open seven days a week, and parents/guardians were encouraged to bring their children to the clinic whenever they were ill. After hours, care was available through the adjacent hospital paediatric ward. Children presenting with new medical problems underwent a standardized medical evaluation using algorithms to guide therapy for illnesses. Medications with anti-malarial activity were avoided for the treatment of non-malarial illnesses. Monthly assessments were done in the study clinic to ensure compliance with study protocols, measure adherence to LLINs through recall of LLIN use in the prior evening, and perform routine blood smears. Study participants were withdrawn for: 1) movement out of the study area, 2) inability to be located for >60 consecutive days, 3) withdrawal of informed consent, 4) inability to adhere to the study schedule and procedures, or, 5) inability to tolerate the drugs used for malaria treatment.

### Malaria diagnosis and management

Subjects who presented with a documented fever (tympanic temperature ≥38.0°C) or history of fever in the previous 24 hours had blood obtained by finger prick for a thick smear. If the thick smear was positive for malaria parasites, the patient was diagnosed with malaria regardless of parasite density. At the time of their first episode of uncomplicated malaria, study participants ≥4 months and ≥5 kg were randomly assigned to receive open-label artemether-lumefantrine (AL) or dihydroartemisinin-piperaquine (DP). Each first daily dose of study drugs was directly observed at the study clinic. Study participants received the assigned treatment for all subsequent episodes of uncomplicated malaria. Episodes of uncomplicated malaria in children less than four months of age or weighing <5 kg as well as episodes of complicated malaria were treated with quinine. Clinical treatment failures occurring within 14 days of initiation of therapy for uncomplicated malaria were treated with quinine. Those occurring within 14 days of initiation of therapy with quinine were treated with quinine plus clindamycin. All children with malaria were followed up on days 1, 2, 3, 7, 14, 21, and 28 following diagnosis. Thick blood smears were collected on all malaria follow-up days apart from day 1 [20].

### Laboratory methods

Thick blood smears were stained with 2% Giemsa for 30 min. Parasite densities were calculated from thick smears by counting the number of asexual parasites per 200 leukocytes (or per 500 leukocytes, if the count was <10 asexual parasites/200 leukocytes), assuming a leukocyte count of 8,000/μl. A blood smear was considered negative when the examination of 100 high power fields did not reveal asexual parasites. For quality control, all slides were read by a second reader, and discrepancies were settled by a third reader. Laboratory technicians were blinded to the study participants’ treatment assignments.

### Statistical methods

Data were double entered into an Access database. Data analysis was done using Stata version 11 (Stata Corp, College Station, TX, USA). The observation period began one day after enrolment and ended when the child turned four years of age or on the day the child was prematurely withdrawn from the study.

Incident episodes of malaria were defined as all treatments not preceded by another treatment in the prior 14 days. The incidence of malaria was calculated as the number of episodes per person years at risk. Asymptomatic parasitaemia was defined as a positive routine blood smear in the absence of fever that was not followed by the diagnosis of malaria in the subsequent seven days. As asymptomatic parasitaemia was assessed using active surveillance, the monthly prevalence of asymptomatic parasitaemia was ascertained for each month of observation. Geometric mean parasite densities were calculated for symptomatic malaria and asymptomatic parasitaemia.

Generalized additive regression with smoothing splines were used to visualize the effects of age, calendar time, and season on the incidence of malaria and prevalence of asymptomatic parasitaemia [21]. Inference was obtained using bootstrapping at the level of an individual with 1,000 replicates. The relative risk of incident malaria and prevalent asymptomatic parasitaemia by age, calendar time and season was calculated using generalized estimating equations with robust standard errors [22].

### Ethical approval

Informed consent was obtained from the parent or guardian of all study participants. The study protocol was approved by the Uganda National Council of Science and Technology and the institutional review boards of the University of California, San Francisco, Makerere University and the Centers for Disease Control and Prevention.

## Results

### Study profile and descriptive statistics

A total of 100 children aged between six weeks and <10 months of age (median 5.5 months) were enrolled between August 2007 and January 2008. Children were followed for a median of 3.5 years. Of 100 children enrolled, 96 were followed through one year of age, 88 through two years of age, 83 through three years of age, and 79 through four years of age. Children were prematurely withdrawn for the following reasons: 13 moved out of the study area, three were unable to comply with study procedures, two withdrew consent, one had treatment failure following quinine + clindamycin, one was unable to be re-administered study drugs due to drug toxicity, and one died. At the time of monthly routine assessment, 98% of study participants were reported to have slept under an LLIN the prior evening (Table 1).

**Table 1:** Descriptive statistics of study cohort

**Characteristic**	**Findings**
Number of children enrolled	100
Median age at enrolment in months (range)	5.52 (1.50-9.93)
Female, n (%)	40 (40%)
Rural residence, n (%)	76 (76%)
Number of children who reached four years of age, n (%)	79 (79%)
Median years of follow-up (IQR)	3.46 (3.24-3.66)
Proportion who reported sleeping under an LLIN* (%)	3,934/4,008 (98%)
Antimalarial group children were randomized to, n (%)	
DP	51 (51%)
AL	42 (42%)
Never randomized	7 (7%)
Total incident episodes of malaria**	1,633
Uncomplicated malaria treated with DP	847
Uncomplicated malaria treated with AL	778
Uncomplicated malaria treated with quinine for age <4 months	2
Quinine for complicated malaria	6
Treatments for malaria within 14 days of prior episode	12
Quinine for early treatment failure following AL	5
Quinine for recurrent malaria on day 14 following AL	2
Quinine/clindamycin for early treatment failure following quinine	3
Quinine/clindamycin for recurrent malaria days 4–14 following quinine	2
Median malaria incidence through four years of age per person years (IQR)	5.44 (3.18-7.00)
Total episodes of asymptomatic parasitaemia	186
Monthly prevalence of asymptomatic parasitaemia	5.0%

Note: IQR, interquartile range; LLIN, long-lasting insecticide treated bed net; DP, dihydroartemisinin-piperaquine; AL, artemether-lumefantrine.

* Assessed at the time of monthly routine visits to the clinic.

** All treatments for malaria not proceed by another treatment in the prior 14 days.

### Treatments for malaria

A total of 1,633 incident cases of malaria were observed. Only six of 1,633 (0.4%) incident cases were complicated (all so-classified based on a single convulsion). There were 12 treatment failures within 14 days of treatment: five early treatment failures associated with complicated malaria following treatment with AL due to single convulsions (two) or severe anaemia (three, all in same child); two episodes of recurrent malaria 14 days after treatment with AL; and five treatments with quinine + clindamycin following treatment with quinine. The only death occurred at home in a 35-month-old boy one day after he was diagnosed with his 27^th^ incident episode of malaria. Of note, this child had three prior episodes of severe anaemia two days following initiation of treatment with AL, representing the only episodes meeting criteria for severe malaria in the entire cohort. Considering parasite clearance, 94.9% and 99.8% of patients had a negative blood smear two and three days following initiation of therapy, respectively.

### Malaria incidence

**Figure 1:**
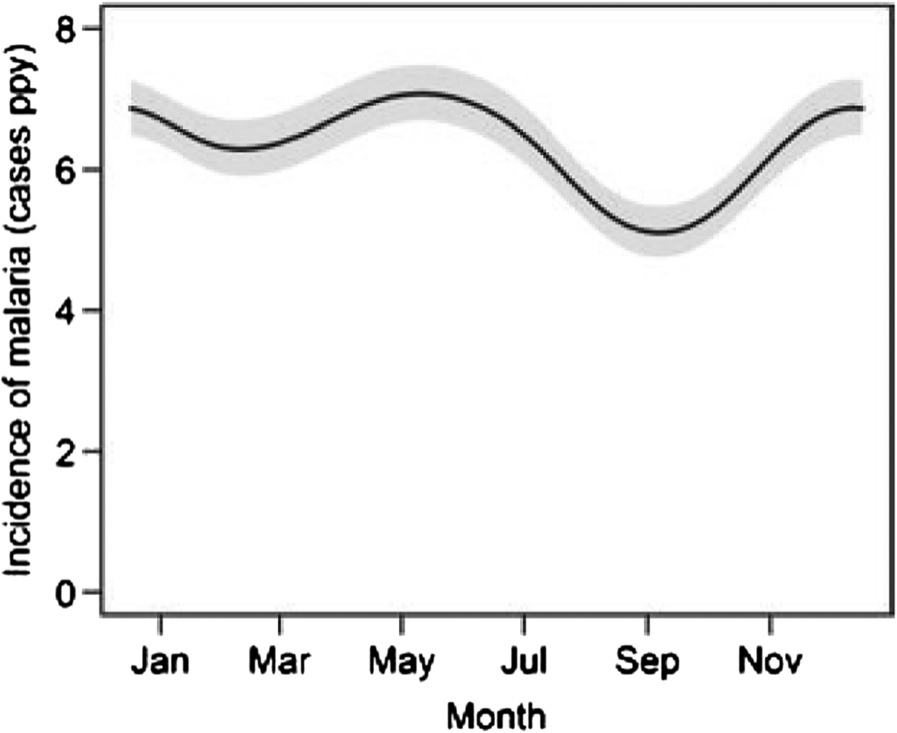
**Predictors of malaria incidence - Malaria incidence by season.** Predictors of malaria incidence visualized using a generalized additive regression model with smoothing splines. The model included season, calendar date, and age. Standard errors are indicated by shaded areas and were generated by bootstrapping with 1,000 replicates. Absolute incidence values for each of the three variables were calculated using median values for the other two variables.

**Figure 2:**
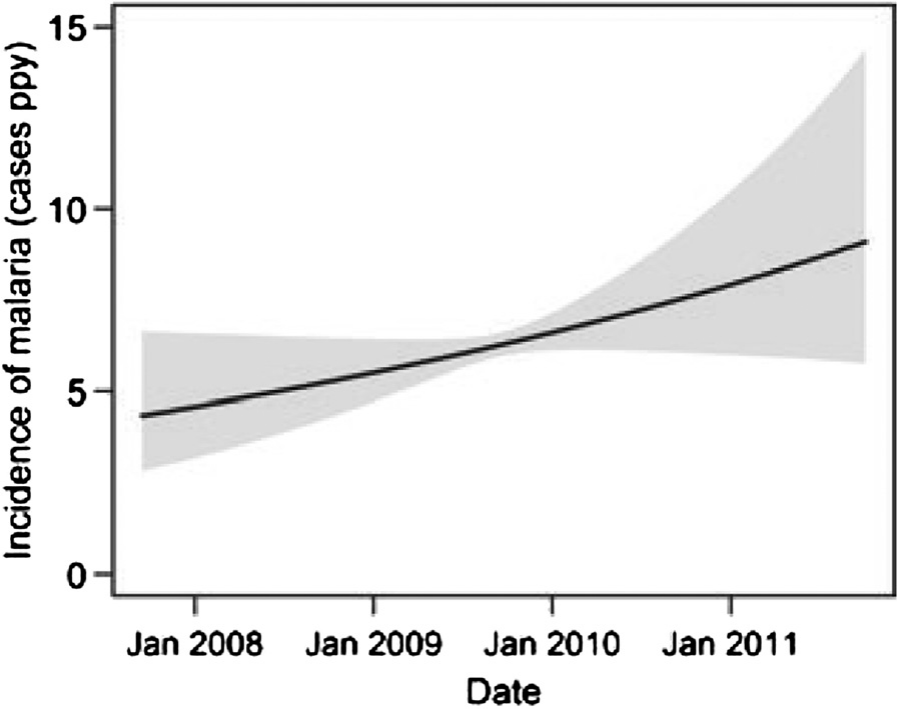
**Predictors of malaria incidence - Malaria incidence by calendar date.** Predictors of malaria incidence visualized using a generalized additive regression model with smoothing splines (see details Figure 1).

**Figure 3:**
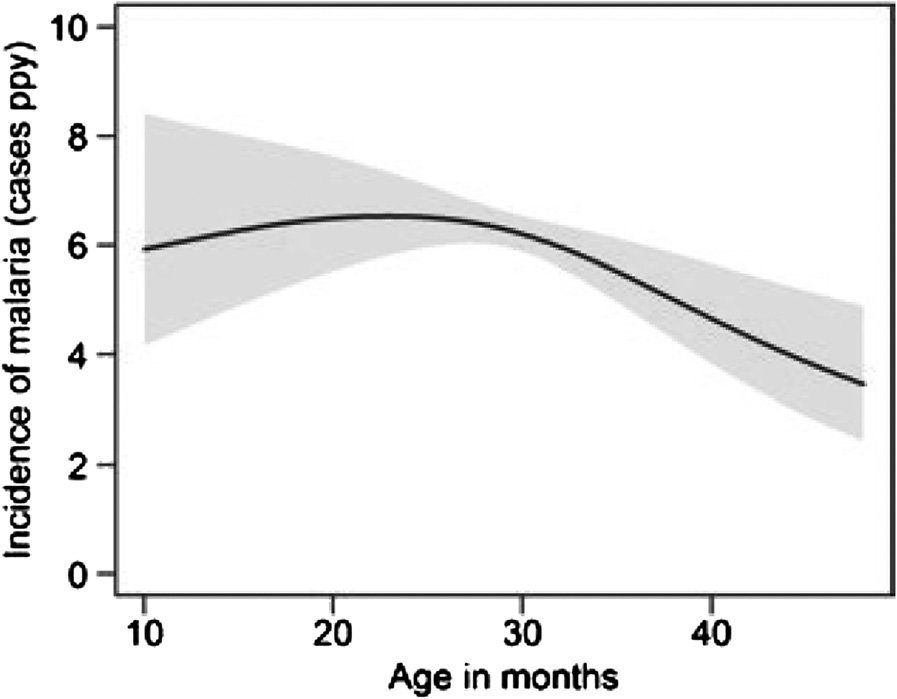
**Predictors of malaria incidence - Malaria incidence by age.** Predictors of malaria incidence visualized using a generalized additive regression model with smoothing splines (see details Figure 1).

The median incidence of malaria was 5.4 episodes PPY (Table 1), with 25% of children having an incidence >7 episodes PPY. Only five children were never diagnosed with malaria and four of these were followed for <6 months. The incidence of malaria was high throughout the year, with two mild seasonal peaks (Figure 1). After adjusting for age and calendar year, the risk of malaria was 28% higher from Nov-Jan (95% CI 14-43%, *P <* 0.001) and 26% higher from Apr-Jul (95% CI 14-40%, *P* < 0.001) compared to the incidence from Aug-Oct (Table 2). The incidence of malaria increased with time over the four years of observation (Figure 2). After adjusting for age and seasonality, the risk of malaria was 52% higher in 2011 compared to 2008 (95% CI 10-109%, *P* = 0.01, Table 2). The incidence of malaria was stable from 10 to <30 months of age, then gradually decreased from 30 to 48 months of age (Figure 3). After adjusting for calendar year and seasonality, the incidence of malaria was 36% lower in children 42 to <48 months of age compared to those 10 to <18 months of age (95% CI 14-52%, *P* = 0.003).

**Table 2:** Factors associated with malaria incidence

**Risk factor**	**Categories**	Relative Risk* (95% CI)	**p-value**
Season	August-October	1.0 (reference)	-
	February-March	1.03 (0.92-1.16)	0.56
	November-January	1.28 (1.14-1.43)	<0.001
	April-July	1.26 (1.14-1.40)	<0.001
Year	2008	1.0 (reference)	-
	2009	1.15 (0.97-1.36)	0.10
	2010	1.42 (1.11-1.82)	0.005
	2011	1.52 (1.10-2.09)	0.01
Age	10- < 18 months	1.0 (reference)	-
	18- < 24 months	1.05 (0.92-1.19)	0.48
	24- < 30 months	1.09 (0.91-1.30)	0.34
	30- < 36 months	0.97 (0.79-1.19)	0.77
	36- < 42 months	0.78 (0.61-1.00)	0.05
	42- < 48 months	0.64 (0.48-0.86)	0.003

Note: CI, confidence interval.

### Asymptomatic parasitaemia

**Figure 4:**
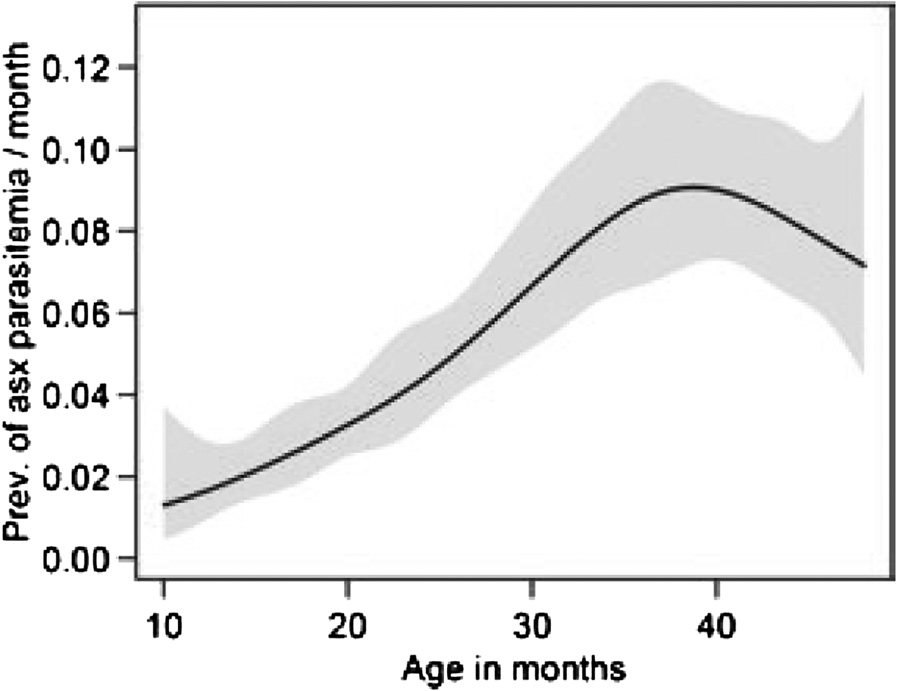
**Prevalence of asymptomatic parasitaemia.** Prevalence of asymptomatic parasitaemia by age modelled by generalized additive regression. Standard error is indicated by the shaded area and was generated by bootstrapping with 1,000 replicates.

Of 6,951 blood smears obtained outside of the first 14 days of malaria follow-up, 475 demonstrated parasitaemia in children without fever. Of these, 186 met the specified definition of asymptomatic parasitaemia, as 289 were followed by malaria within seven days. Overall, the monthly prevalence of asymptomatic parasitaemia was 5.0% (Table 1.) Asymptomatic parasitaemia was uncommon throughout the study, peaking at ~9% at 39 months of age (Figure 4). The monthly risk of asymptomatic parasitaemia was significantly higher in children 36 to <48 months of age (8.3%) compared to those 10 to <18 months of age (2.5%) (RR 4.4, 95% CI 2.6-7.5, *P* < 0.001). Significant heterogeneity in the prevalence of asymptomatic parasitaemia was observed, with 10% of children having nearly 55% of the total episodes and 45% of children having no episodes of asymptomatic parasitaemia during follow-up (P < 0.001 overdispersed compared with Poisson). Asymptomatic parasitaemia was more common in children with the most malaria; all children having more than 10 episodes of asymptomatic parasitaemia were in the highest quartile of incident malaria, and only one child with less than two episodes of malaria per year had an episode of asymptomatic parasitaemia.

### Effect of age on parasite density

The geometric mean parasite density was assessed by six-month age increments, stratified by symptomatic malaria and asymptomatic parasitaemia. The geometric mean parasite density was significantly higher during malaria episodes than during episodes of asymptomatic parasitaemia (22,200/μL *vs* 1,900/μL, respectively; *P* < 0.0001). There was no significant trend for parasite densities for either malaria or asymptomatic parasitaemia over age (Table 3).

**Table 3:** Geometric mean parasite densities (GMPD) by age and clinical manifestation

**Clinical manifestation**	**Asexual parasites/μL stratified by age in months**						p-value*	**All ages**	p-value**
	**10- < 18**	**18- < 24**	**24- < 30**	**30- < 36**	**36- < 42**	**42- < 48**			
Symptomatic malaria	20916	24177	20912	24631	20329	22813	.938	22216	ref
Asymptomatic parasitaemia	1545	1336	2018	1948	2010	1928	.905	1901	<0.001

* Comparisons between log-transformed parasite densities by age groups using generalized estimating equations.

** Comparison between GMPD of malaria *vs* asymptomatic parasitaemia using generalized estimating equation.

## Discussion

In this longitudinal cohort of 100 children given LLINs and ACT and residing in an area of known high transmission intensity in eastern Uganda, a remarkably high incidence of malaria was observed, peaking at 6.5 episodes per child per year at 25 months of age. Importantly, the vast majority (99.7%) of malaria cases were uncomplicated. The incidence declined as children reached three years of age, suggesting the development of natural immunity to malaria [23], although children on average still had four episodes per year at four years of age, and asymptomatic parasitaemia, a finding typically associated with anti-malarial immunity, was observed in less than 10% of individuals. Perhaps most concerning, in the setting of near universal use of LLINs to limit mosquito exposure and ACT to effectively treat malaria, the incidence of malaria was very high throughout this study and rose 52% from 2008 through 2011.

Many reports across Africa have shown significant declines in malaria-related deaths and hospitalizations over the past decade, and it is routinely suggested that malaria-control interventions - including usage of LLINs and ACT - are responsible for these declines [4-8]. However, few of these studies were done with longitudinal cohorts in high-endemicity settings. Contrasting with many reports, this study and recent reports from Malawi, Uganda, and Senegal showed a lack of decline in malaria incidence [5,9,10].

The reason for a lack of decline, and indeed a significant increase in the incidence of malaria in Tororo is unknown. The increase may be due to changes in drug resistance, vector species, insecticide resistance, and/or climate/rainfall, leading to increases in exposure to malaria vectors. Considering drugs, AL and DP have both shown outstanding efficacy in numerous recent studies, and there is no convincing evidence of resistance to these agents in Africa. Although we did not collect detailed information on mosquito species or biting habits, preliminary data from the East African International Centers of Excellence in Malaria Research (ICEMR) estimates the EIR in Tororo to be 379 infective bites PPY (unpublished data from CDC light traps collected between Oct 2011 and Sep 2012), near the EIR of 562 infective bites PPY estimated by human landing catches in 2002 [16]. The majority of mosquitoes collected during this period were *Anopheles gambiae* complex (93.5%), as found in 2002 [16]. Notably, population based surveys in this area have revealed significant increases in LLIN coverage in Tororo over the past six years, with the proportion of children <5 years of age sleeping under a LLIN increasing from 13% in 2006 [24] to 42% in 2009 [25] and 62% in 2012 (East African ICEMR, unpublished). However, several recent reports have also shown increasing resistance of anopheline mosquitoes to pyrethroid insecticides in Tororo. Prevalence of the knockdown resistance (KDR) *L1014S* mutation has increased from 29% in 2002 to 75% in 2008 in mosquitoes collected from Tororo [26,27], suggesting that the effectiveness of LLINs may be waning in this area, as also seen in other parts of Africa [10,28]. In these settings, additional control measures - including indoor residual spraying [29], larvicides and other vector control measures [30], and chemoprevention [31], will be required.

Importantly, even though more than 1,600 cases of malaria were diagnosed in the 100 children enrolled in this cohort, remarkably few cases of malaria were associated with complications. Only six episodes met criteria for complicated malaria, and all these cases were due to a single convulsion (danger sign) and did not meet criteria for severe malaria. There were no cases of cerebral malaria or respiratory distress. This result is in sharp contrast to historical reports suggesting that 2% of all clinical attacks of malaria are severe [32]. Consistent with earlier observations from a cohort study in Uganda conducted in a lower transmission setting [33,34], these findings suggest that malaria-related morbidity can be greatly limited with prompt access to appropriate diagnosis and highly effective treatment. Early treatment failures were also exceedingly rare, consistent with prior findings that ACT is very efficacious [35-38]. Of 12 treatment failures within 14 days of prior therapy, there were three cases of severe anaemia, all in the same child. The unfortunate death of this two-year old boy, who had had 27 prior episodes of malaria, is clear evidence that, even with prompt diagnosis and therapy, malaria remains a dangerous disease in need of improved control measures.

As others have reported, children in this cohort appeared to acquire anti-disease immunity as they aged. The incidence of malaria declined in children after 30–36 months of age, and asymptomatic parasitaemia was more common in older children [23]. However, given the degree of exposure and high incidence in the cohort, the natural acquisition of anti-malarial immunity in this cohort was remarkably slow. A surprising finding was the low prevalence of asymptomatic parasitaemia in this cohort - 5% based on monthly microscopy. In contrast, parasite prevalence rates from eastern Uganda in the 2009 malaria indicator survey were nearly 40% in children <5 [25], and in a similarly aged cohort from Kampala, Uganda, a lower transmission setting, the prevalence of asymptomatic parasitaemia was much higher (17%) [39]. Given a lack of standard diagnostic criteria, asymptomatic parasitaemia described in this report accounted for whether or not children were in a pre-symptomatic period, excluding any child that developed malaria within seven days [40]. Although varying definitions may explain some of these differences, the significantly low prevalence of asymptomatic infection observed here suggests that prompt and repeated treatments with highly effective ACT may alter the acquisition of anti-parasite immunity, limiting infections to symptomatic disease in young children. Further studies addressing the impact of ACT on the development of anti-malarial immunity are needed.

There were several limitations to this study. The cohort was enrolled by convenience sampling, limiting the generalizability of the findings. Although the analysis attempted to adjust for calendar time, children enrolled early in the study may have differed from those enrolled later. Detailed information was also unavailable regarding individual- and household-level exposure. A further limitation was the assessment for LLIN use by self-report, and not by directly observed LLIN usage, which may have overestimated LLIN coverage [41]. Finally, children received treatment for malaria if they presented with fever and any parasitaemia. If non-malarial causes of fever were the aetiology of some of these presentations, malaria incidence may have been overestimated in this cohort. However, the geometric mean parasite densities of malaria episodes were significantly higher than those of episodes of asymptomatic parasitaemia, suggesting that malaria contributed to symptoms in most cases where anti-malarial therapy was given.

## Conclusions

In conclusion, despite reports of decreasing malaria morbidity and mortality across many parts of Africa, the incidence of malaria continues to be very high in Tororo, Uganda, even in a setting with LLINs and ACT, and this incidence appears to be rising. Thus, additional malaria control interventions among young children living in high transmission settings are needed. Fortunately, with prompt diagnosis and effective treatment, malaria-related complications can be limited even in high transmission settings.

## Competing interests

The authors declare that they have no competing interests.

## Authors’ contributions

GD, JT, PJR, and MRK conceived and designed the study. MKM, AK, and EA participated in data collection. PJ, BG, and GD participated in the data analysis. All authors participated in the writing of the manuscript, read and approved the final manuscript.
